# *Vibrio splendidus* flagellin C binds tropomodulin to induce p38 MAPK-mediated p53-dependent coelomocyte apoptosis in Echinodermata

**DOI:** 10.1016/j.jbc.2022.102091

**Published:** 2022-05-30

**Authors:** Fa Dai, Ming Guo, Yina Shao, Chenghua Li

**Affiliations:** 1 State Key Laboratory for Quality and Safety of Agro-products, Ningbo University, Ningbo, China; 2 State-Province Joint Laboratory of Marine Biotechnology and Engineering, Ningbo University, Ningbo, China; 3 Laboratory for Marine Fisheries Science and Food Production Processes, Qingdao National Laboratory for Marine Science and Technology, Qingdao, PR China

**Keywords:** flagellum, tropomodulin, *Vibrio splendidus*, *Apostichopus japonicus*, apoptosis, FliC, lagellin C, PAMP, pathogen-associated molecular pattern, PBST, PBS containing Tween-20, PRR, pattern recognition receptor, rFliC, recombinant FliC, siTmod, siRNA targeting AjTmod, TLR, Toll-like receptor

## Abstract

As a typical pathogen-associated molecular pattern, bacterial flagellin can bind Toll-like receptor 5 and the intracellular NAIP5 receptor component of the NLRC4 inflammasome to induce immune responses in mammals. However, these flagellin receptors are generally poorly understood in lower animal species. In this study, we found that the isolated flagellum of *Vibrio splendidus* AJ01 destroyed the integrity of the tissue structure of coelomocytes and promoted apoptosis in the sea cucumber *Apostichopus japonicus*. To further investigate the molecular mechanism, the novel intracellular LRR domain-containing protein tropomodulin (AjTmod) was identified as a protein that interacts with flagellin C (FliC) with a dissociation constant (*K*_*d*_) of 0.0086 ± 0.33 μM by microscale thermophoresis assay. We show that knockdown of AjTmod also depressed FliC-induced apoptosis of coelomocytes. Further functional analysis with different inhibitor treatments revealed that the interaction between AjTmod and FliC could specifically activate p38 MAPK, but not JNK or ERK MAP kinases. We demonstrate that the transcription factor p38 is then translocated into the nucleus, where it mediates the expression of p53 to induce coelomocyte apoptosis. Our findings provide the first evidence that intracellular AjTmod serves as a novel receptor of FliC and mediates p53-dependent coelomocyte apoptosis by activating the p38 MAPK signaling pathway in Echinodermata.

Innate immunity is the first line of defense against invasive pathogens, and it is activated rapidly after pathogen invasion (1). The innate immune system has gradually become known for its specificity in resisting bacterial infection. It can specifically recognize substances from different sources through a series of receptors and cause signal cascade transduction to promote immune defense (2), which relies on the detection of pathogen-associated molecular patterns (PAMPs) by special pattern recognition receptors (PRRs).

Flagella are the main motor organs of bacteria and have multiple biological functions in pathogenicity (3, 4, 5, 6). As the major subunit of the flagellum, flagella C (FliC) has attracted much attention as a regular PAMP (7). FliC was initially considered a virulence factor of *Streptococcus typhimurium* that did not present any immune-stimulating effects in mice (8, 9). Subsequently, the proinflammatory function mediated by FliC was reported in different bacteria (10, 11). Studies have found that *Salmonella* FliC is an effective inducer of the expression of cytokines TNF-α, IL-1β, IL-6, and IL-10, and this proinflammatory phenomenon was reported to involve the interaction between FliC and immune cell receptors (12). The interaction between receptors and FliC was identified as being mediated by Toll-like receptor (TLR)5 by Hayashi *et al*. (13). The interaction between FliC and TLR5 and its effect on immune stimulation have also been reported in other studies (14, 15, 16). Subsequently, a variety of FliC receptors have been found in different species, among which TLR and Nod-like receptor (NLR) family receptors are the most prominent in FliC-mediated pathogen-host receptor recognition (17, 18, 19, 20, 21).

The recognition of TLR and NLR receptors and FliC was found to be dependent on the LRR domain (22); receptor binding to the D1 and D2 regions of FliC plays a key role in this interaction and was found to be essential for activating proinflammatory responses (9, 23). LRR domains established from two or more tandem LRRs were used for protein–protein interaction studies and then were found to participate in signal transduction, tissue development, cell adhesion, and many other biological processes (24, 25, 26, 27). As the basic recognition module of typical PRRs, 50 disease-related LRR domain-containing proteins have been identified in a wide range of animals, including TLRs, NLRs, small leucine-rich repeat proteoglycans, tropomodulin (Tmod), and F-box/LRR-repeat proteins (28). Tropomodulin is the only protein known to cap the pointed end of tropomyosin-coated actin filaments, which typically consist of a tropomyosin-binding helix region at the N-terminus, an actin-binding region, and LRR regions at the C-terminus (29, 30). Tmods play an important role in the regulation of actin dynamics and cytoskeletal structure, which further determines cell morphology, cytomechanics, contraction, and dendritic processes (31, 32). Moreover, Tmod1 and Tmod3 were found to be closely related to liver cancer (33, 34). Knockout of Tmod1 in dendritic cells resulted in NF-κB and p38-MAPK pathway inhibition, which downregulated the expression of costimulatory molecules and proinflammatory cytokines (35). Several studies revealed that Tmods levels were significantly upregulated in peripheral blood mononuclear cells or macrophages of patients (36). These results indicated that Tmods were expressed in immune cells and possibly involved in regulating immune responses.

Skin ulcer syndrome caused by *Vibrio splendidus* was reported to cause higher than 80% mortality and lead to economic losses of 30% in *Apostichopus japonicus* culture, an important aquaculture species in China and Japan (37, 38). Understanding the interaction between pathogen virulence and the host immune response is crucial for establishing an effective disease control strategy. However, the receptor for the *V. splendidus* FliC protein and its mediation of the immune response are largely unknown to our knowledge. In this study, an AJ01 FliC interactive protein was first identified and characterized as intracellular AjTmod using sea cucumber as a model. The interaction between AjTmod and FliC was dependent on the LRR domain of AjTmod. We further confirmed that the FliC–AjTmod complex could specifically activate the p38-MAPK pathway and promote p38 transport into the nucleus, which mediated proapoptotic factor p53-dependent coelomocyte apoptosis. These findings provide insights into the mechanisms of the flagellum-mediated immune response, which advance our understanding of pathogen–host interactions.

## Results

### Flagellum of *V. splendidus* AJ01 could mediate the immune response in *A. japonicus*

**Figure 1:**
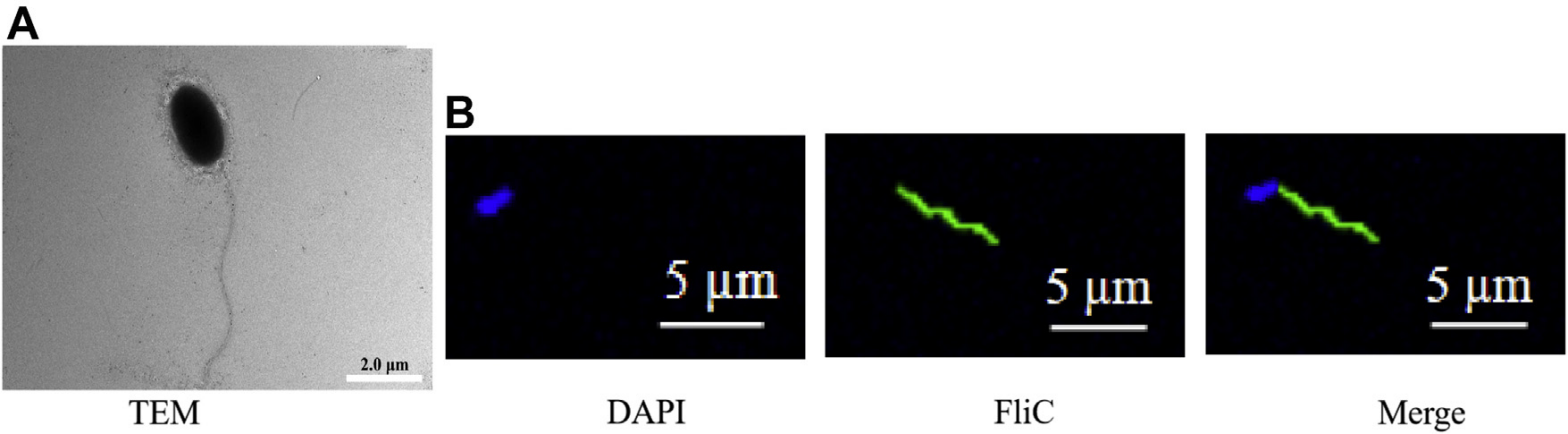
**Morphological observations of the *Vibrio splendidus* AJ01 flagellum.***A*, transmission electron micrograph of AJ01. The scale bars represent 2 μm. *B*, fluorescence staining of AJ01. DAPI was used to stain the nucleus, and *green fluorescence* was used to stain the flagellum. The images were taken under 40× magnification. The scale bars represent 10 μm.

**Figure 2:**
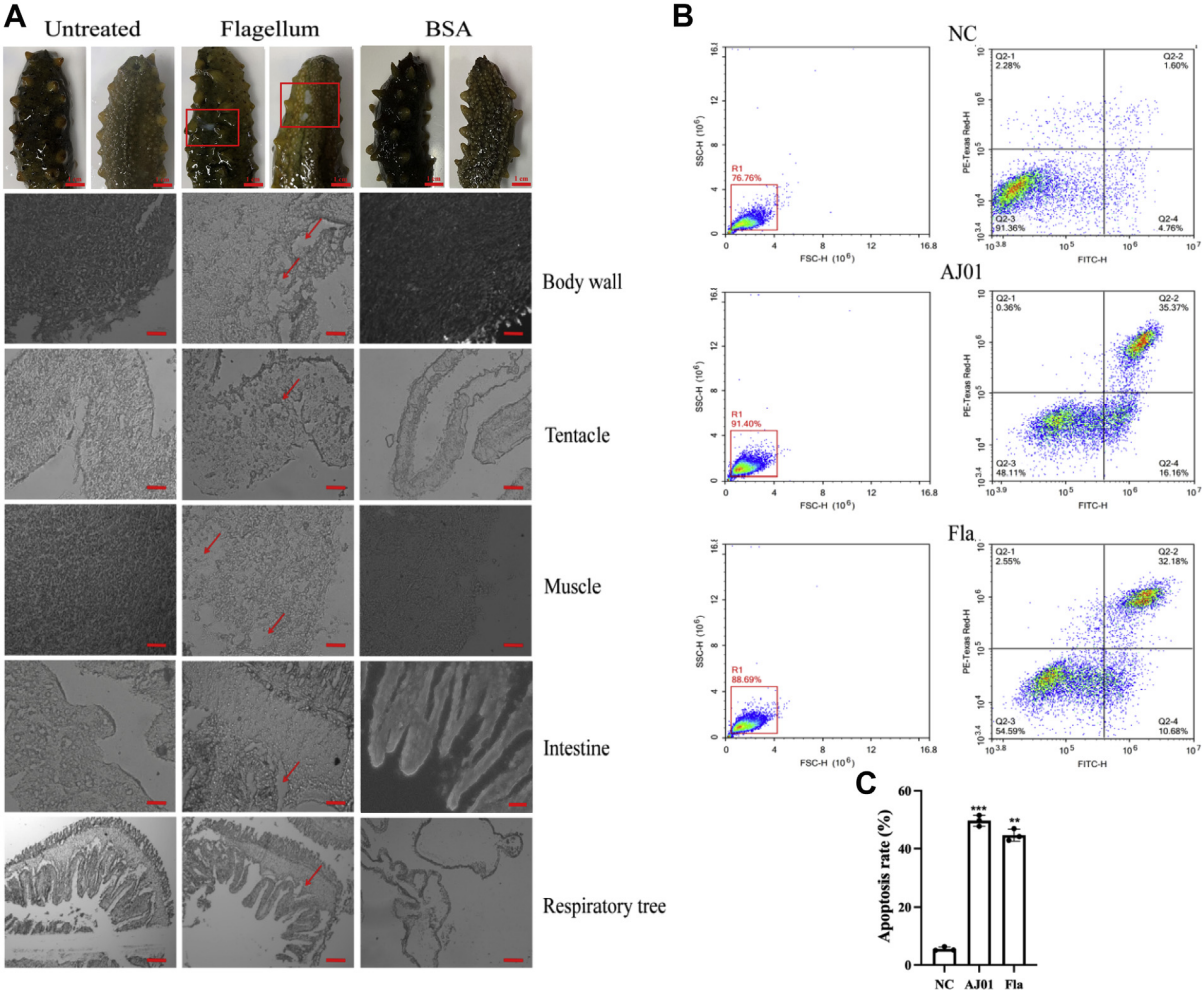
**Immune function of the AJ01 flagellum in *Apostichopus japonicus*.***A*, tissue structure changes in sea cucumbers at 24 h after 100 μg flagellum treatment. The *first column* presents healthy sea cucumbers without any treatment that served as the blank control. The *second column* presents sea cucumbers treated with 100 μg flagellum for 24 h. The *third column* presents sea cucumbers treated with 100 μg BSA for 24 h. The symptoms of skin ulceration of sea cucumbers are indicated with *red rectangles*. *Red arrows* indicate obvious tissue damage. The scale bars represent 200 μm. *B*, flow cytometry analysis of the changes in coelomocyte apoptosis at 24 h after AJ01 (10^7^ CFU/ml) and flagellum treatment (100 μg). *C*, statistical analysis of the change in coelomocyte apoptosis from (*B*) (n = 3). ∗∗*p* < 0.01, ∗∗∗*p* < 0.001. BSA, bovine serum albumin.

FliC is a surface-associated protein and the most easily accessible microbial antigen recognized by the host immune system, which can lead to host tissue lesions, apoptosis, and necrosis (39, 40). To explore the immune response triggered by the flagellum in *V. splendidus* AJ01, we first visualized the existence of flagellum using a transmission electron microscope. The thallus of AJ01 was short and punctate and approximately 1 to 2 μm in length, and the flagellum could reach approximately 6 to 8 μm (Fig. 1*A*). FliC was the major subunit of the flagellum; hence, we prepared an antiserum against a recombinant FliC protein (rFliC) to identify the flagellum of AJ01. After FliC antiserum incubation, the flagellum presented an obvious green signal with a similar size to that in Figure 1*A* (Fig. 1*B*). Subsequently, we extracted the AJ01 flagellum (100 μg) and examined its effects on tissue morphology and coelomocyte apoptosis in sea cucumber. After injection of the flagellum into the sea cucumber coelome for 24 h, obvious symptoms of skin ulceration were observed, while this symptom was not observed in bovine serum albumin (BSA)-treated and untreated sea cucumbers. We next performed a histological analysis to further detect the tissue structure changes in the body walls, tentacles, muscles, intestines, and respiratory trees of the three groups. The results revealed that the integrity of the tissue structure was destroyed and that tissue connections were loosened in the five examined tissues. After flagellum treatment, the muscle fiber cells sloughed, and dense muscle fibers appeared as holes, especially in the body wall and muscle tissue. However, the two control groups exhibited no obvious tissue morphology changes (Fig. 2*A*). Furthermore, flow cytometry was applied to test the changes in apoptosis levels in coelomocytes under the same treatment conditions. The results showed that the flagellum could promote significantly more coelomocyte apoptosis levels than that of the control treatment (Fig. 2, *A* and *B*). Accordingly, these results confirmed that the flagellum served as an immunogen involved in the immune response of *A. japonicus*.

### AjTmod is an AJ01 FliC-interacting protein

**Figure 3:**
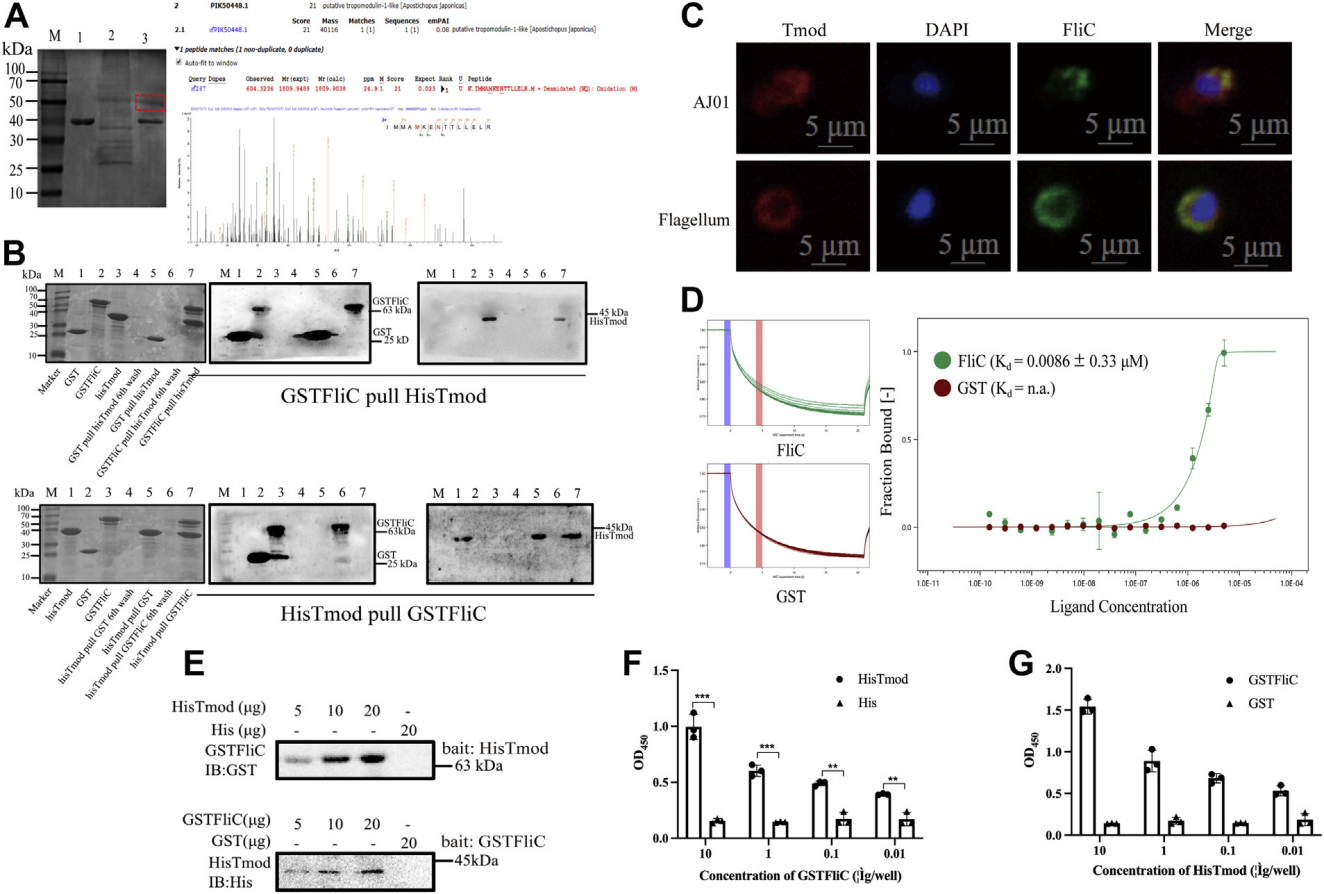
**FliC specifically binds AjTmod.***A*, differential bands were obtained by HisFliC pull-down and identified as AjTmod by mass spectrometry. M: protein marker; Lane 1, purified rFliC; Lane 2, sea cucumber coelomocyte lysates; Lane 3, rFliC and binding protein eluted by Ni-NTA elution buffer. *B*, pull-down assays of the interaction between purified GSTFliC and HisTmod. SDS–PAGE and Western blotting assays to detect GSTFliC and HisTmod interactions are shown. *Upper Panel* M: Protein marker; Lane 1, purified rGST; Lane 2, purified GSTFliC; Lane 3, purified HisTmod; Lane 4, rGST pulled HisTmod sample after six washes; Lane 5, rGST pulled HisTmod elution sample; Lane 6, GSTFliC pulled HisTmod sample after six washes; Lane 7, GSTFliC pulled HisTmod elution sample. *Lower Panel* M: Protein marker; Lane 1, purified HisTmod; Lane 2, purified rGST; Lane 3, purified GSTFliC; Lane 4, HisTmod-pulled rGST sample after six washes; Lane 5, HisTmod-pulled rGST elution sample; Lane 6, HisTmod-pulled GSTFliC sample after six washes; Lane 7, HisTmod-pulled GSTFliC elution sample. The first panel in each *row presents* the SDS–PAGE results. The second panel in each *row presents* Western blotting analysis of the GST tag signal performed using a GST-labeled mouse monoclonal antibody. The third panel in each row presents Western blotting analysis of the His tag signal performed using a His-labeled mouse monoclonal antibody. *C*, immunofluorescence of AjTmod and FliC colocalization. *Upper panels* present sea cucumber primary coelomocytes infected with AJ01 (10^7^ CFU/ml), and *lower panels* present primary coelomocytes infected with flagellum (20 μg) using FliC-labeled mouse polyclonal antibody and AjTmod-labeled rabbit polyclonal antibody by laser confocal technology. *Green* and *red* fluorescence represent the expression of FliC and AjTmod, respectively. The second panel in each *row* presents the nuclei stained by DAPI. The fourth panel in each *row* presents the image of the front three panels with digital overlays to visualize the colocalization. The images were taken under 4× lenses. The scale bars represent 5 μm. *D*, binding curves and dissociation constant (*K*_*d*_) of AjTmod with GSTFliC or GST using microscale thermophoresis (MST). The results are expressed as the mean ± SD derived from three independent repeats (n = 3). *E*, confirmation of the FliC–AjTmod interaction by far-western assay. *Upper panel*: Lane 1, 5 μg of HisTomd; Lane 2, 10 μg of HisTomd; Lane 3, 20 μg of HisTomd; Lane 4, 20 μg of rHis tag. *Lower panel*: Lane 1, 5 μg GSTFliC; Lane 2, 10 μg GSTFliC; Lane 3, 20 μg GSTFliC; Lane 4, 20 μg rGST tag. All samples were run on an SDS–PAGE gel and transferred onto a PVDF membrane. The proteins were renatured on the membrane by adding a series of concentrations of guanidine-HCl. rGSTFliC/rHisTmod was added to recognize rHisTmod/rGSTFliC. Attached proteins were detected by GST- and His-labeled antibodies. *F*, recognition of GSTFliC by HisTmod. GSTFliC (10 μg, 1 μg, 0.1 μg, and 0.01 μg per well) was coated in 96-well plates. After the wells were blocked with 5% BSA, 600 ng of HisTmod was added to the well to bind for 3 h at 28 °C. The bound proteins were detected using conventional ELISA with a His-labeled antibody. *G*, recognition of HisTmod by GSTFliC. HisTmod (10 μg, 1 μg, 0.1 μg, and 0.01 μg per well) was coated in 96-well plates. After the wells were blocked with 5% BSA, 600 ng of GSTFliC was added to the wells to bind for 3 h at 28 °C. The bound proteins were detected using conventional ELISA with a GST-labeled antibody. The results were expressed as the mean ± SD derived from three independent repeats. BSA, bovine serum albumin; FliC, flagellin C; rFliC, recombinant FliC.

Considerable evidence indicates that FliC functions as a PAMP in the control of pathogenic infections caused by bacteria by binding to the surface or cytosolic receptor of host cells (41, 42, 43, 44). To identify the candidate interactive protein of flagellin, we used flagellum monomers of FliC from *V. splendidus* AJ01 as the target to isolate the potential interacting protein from sea cucumber coelomocytes. An obvious differential band was detected (red framed) by SDS–PAGE analysis and further characterized as AjTmod by mass spectrometry (Fig. 3*A*). Based on the data, the open reading frame of AjTmod (GenBank no. PIK50448.1) was cloned (Fig. S1). The predicted protein AjTmod possessed a tropomodulin domain located from amino acids 37 to 148 and a typical LRR domain located from amino acids 222 to 328. The expression of *AjTmod* mRNA and protein was significantly induced by *A. japonicus* AJ01 and flagellum treatment (Fig. S2). Next, a reverse pull-down assay was performed to further examine the interaction between AjTmod and FliC. GSTFliC-, HisTmod-, GST-, or His-coated beads were incubated with or without rHisTmod or rGSTFliC proteins and then analyzed by GST-labeled or His-labeled antibodies. GST and His tags were used as controls. As shown in the upper panel of Fig. 3*B*, immobilized GSTFliC fusion protein could specifically bind to soluble HisTmod. As expected, the incubation of rGSTFliC with immobilized rHisTmod also confirmed their specific binding (Fig. 3*B*, lower panel). None of the target proteins were pulled down by the GST or His tag control protein. Furthermore, laser confocal microscopy was employed to visualize the colocalization of FliC and AjTmod in coelomocytes. After coelomocytes were treated with AJ01 or flagellum for 12 h, the coelomocytes were fixed and incubated with mouse FliC and rabbit AjTmod antibodies. A similar distribution pattern of AjTmod (red) and FliC (green) was observed in the cytoplasm (Fig. 3*C*). FliC could be detected in coelomocytes not only in the extracellular matrix but also intracellularly. To better assess their direct interaction, microscale thermophoresis analysis showed that rHisTmod strongly interacted with rGSTFliC with a dissociation constant (*K*_*d*_) of 0.0086 ± 0.33 μM. However, the GST tag protein exhibited no direct binding with rHisTmod (Fig. 3*D*). Far-Western blotting and ELISA results further supported the specific binding between GSTFliC and HisTmod, in which GSTFliC activity was significantly promoted by different concentrations of HisTmod treatment and vice versa (Fig. 3, *E*–*G*).

### AjTmod recognizes FliC through its LRRs

**Figure 4:**
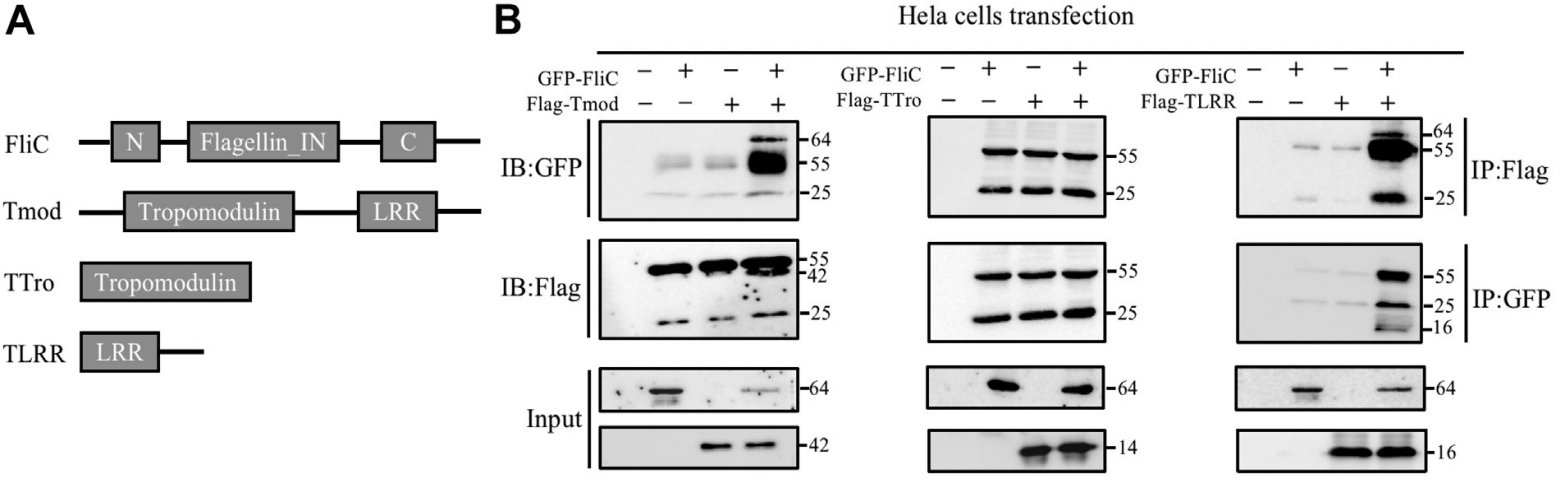
**AjTmod interacts with FliC through its LRR domain.***A*, the corresponding region of Flic and different recombinant plasmids of AjTmod. *B*, immunoprecipitation assay of AjTmod and FliC. HeLa cells were cotransfected with the indicated EGFP-FliC and different recombinant plasmids of AjTmod and cultured for 24 h. The cells were then lysed in lysis buffer and immunoprecipitated with protein A + G for 4 h. GFP- and Flag-labeled antibodies were used for immunoblotting analysis. FliC, flagellin C.

The LRR domain is the basic recognition module of typical PRRs, and its main function is to bind with different ligands, including lipopolysaccharide, nucleic acids, lipids, and bacterial flagellin (24, 25, 45). To confirm whether the LRR domain of AjTmod was responsible for the interaction between AjTmod and FliC, we established and utilized different regions of Flag-fused AjTmod proteins, that is, the LRR domain region from amino acids 664 to 1065 (TLRR), the tropomodulin domain region from amino acids 109 to 444 (TTro), and the complete AjTmod. GFP-FliC fused protein was also generated by the pIRES2-EGFP expression system (Fig. 4*A*). HeLa cells were cotransfected with pIRES2-EGFP-FliC and Flag-tagged Tmod, TLRR, or TTro. The immunoprecipitation results indicated that GFP-FliC could specifically coprecipitate with Tmod or TLRR, not TTro (Fig. 4*B*), which further supported that FliC associates with AjTmod *via* the LRR domain.

### AjTmod is necessary for flagellum-mediated coelomocyte apoptosis

**Figure 5:**
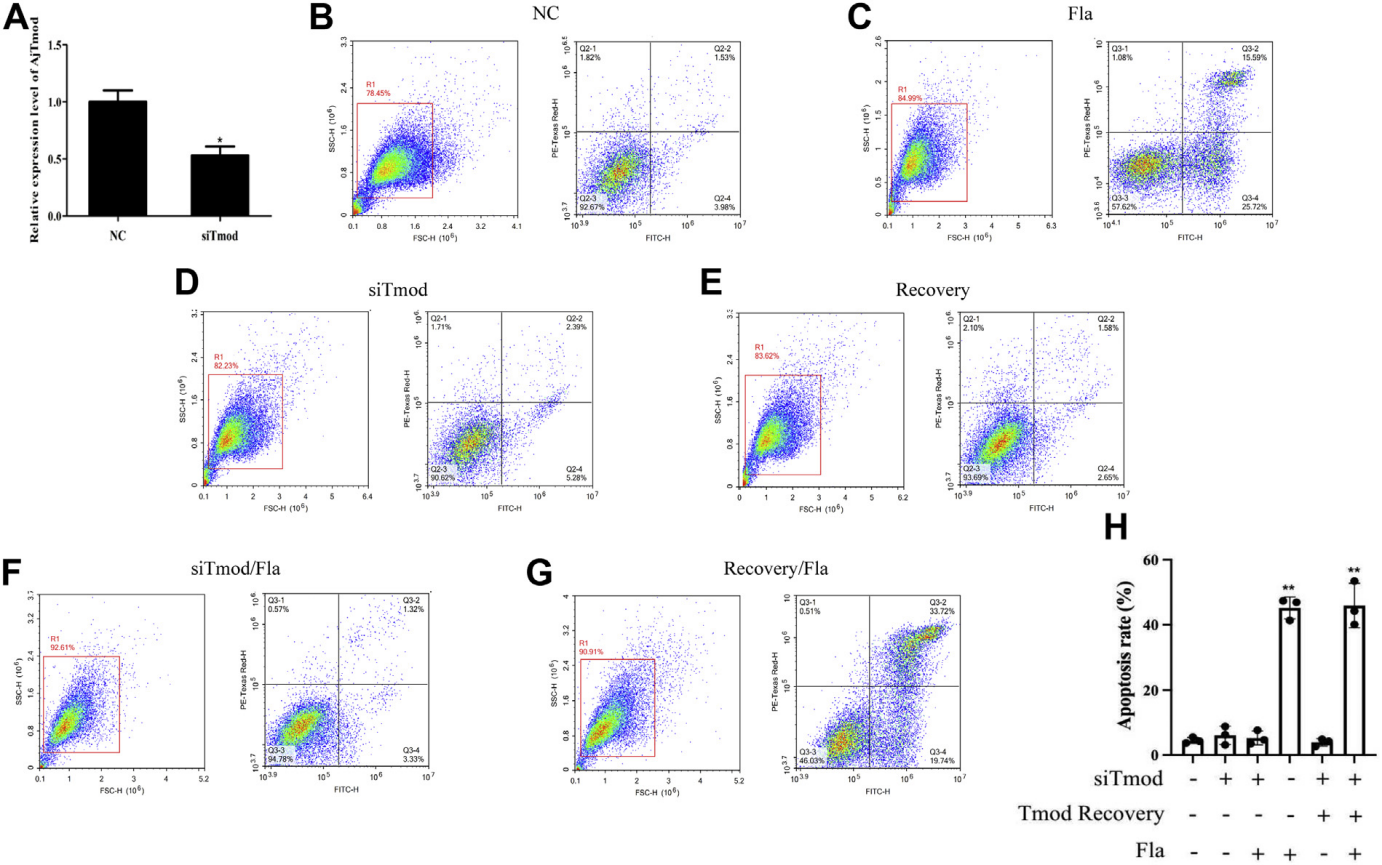
**AjTmod mediates AJ01 flagellum-induced coelomocyte apoptosis.***A*, qRT–PCR analysis of *AjTmod* mRNA expression profiles in coelomocytes post siTmod transfection (n = 3). *B*–*G*, flow cytometry analysis of the changes in coelomocyte apoptosis after siNC transfection (20 μM, 12 h), siTmod transfection (20 μM, 12 h), flagellum treatment (100 μg, 12 h), rTmod recovery after siTmod (100 μg, 12 h), flagellum treatment+siTmod, and rTmod recovery+flagellum treatment. *H*, apoptosis rate of coelomocytes detected under different conditions from (*B*–*G*). Data are the means of three independent experiments and are presented as the means ± SD. siTmod, siRNA targeting AjTmod. ∗*p* < 0.05, ∗∗*p* < 0.01.

Considering that the flagellum positively regulated host coelomocyte apoptosis and directly interacted with AjTmod as described above, we determined whether flagellum-mediated coelomocyte apoptosis was dependent on recurrent AjTmod. A specific siRNA targeting AjTmod (siTmod) was synthesized and transfected into sea cucumbers, which significantly decreased the mRNA level of *AjTmod* at 12 h to 47% of the control group (siNC transfection) levels (Fig. 5*A*). Upon successful knockdown of AjTmod *in vivo*, we next exposed sea cucumbers to rTmod, flagellum, or rTmod+flagellum for another 12 h and detected coelomocyte apoptosis by flow cytometry. Sea cucumbers injected with the same dose of siNC or flagellum were used as the controls. There was no significant difference in coelomocyte apoptosis among the groups treated with siNC (Fig. 5*B*), siTmod (Fig. 5*D*), siTmod+flagellum (Fig. 5*F*), and siTmod+rTmod (Fig. 5*E*), which suggested that AjTmod did not function as a coelomocyte apoptosis regulator alone during coelomocyte apoptosis. Furthermore, the levels of coelomocyte apoptosis in flagellum-treated (Fig. 5*C*) and siTmod+rTmod+flagellum-treated sea cucumbers were significantly higher than those in the siTmod+flagellum-treated group (Fig. 5, *G* and *H*). These data clearly demonstrated that AjTmod acts as an essential interactive protein involved in mediating flagellum-mediated coelomocytes.

### FliC/AjTmod-mediated coelomocyte apoptosis is dependent on the p38-MAPK pathway

**Figure 6:**
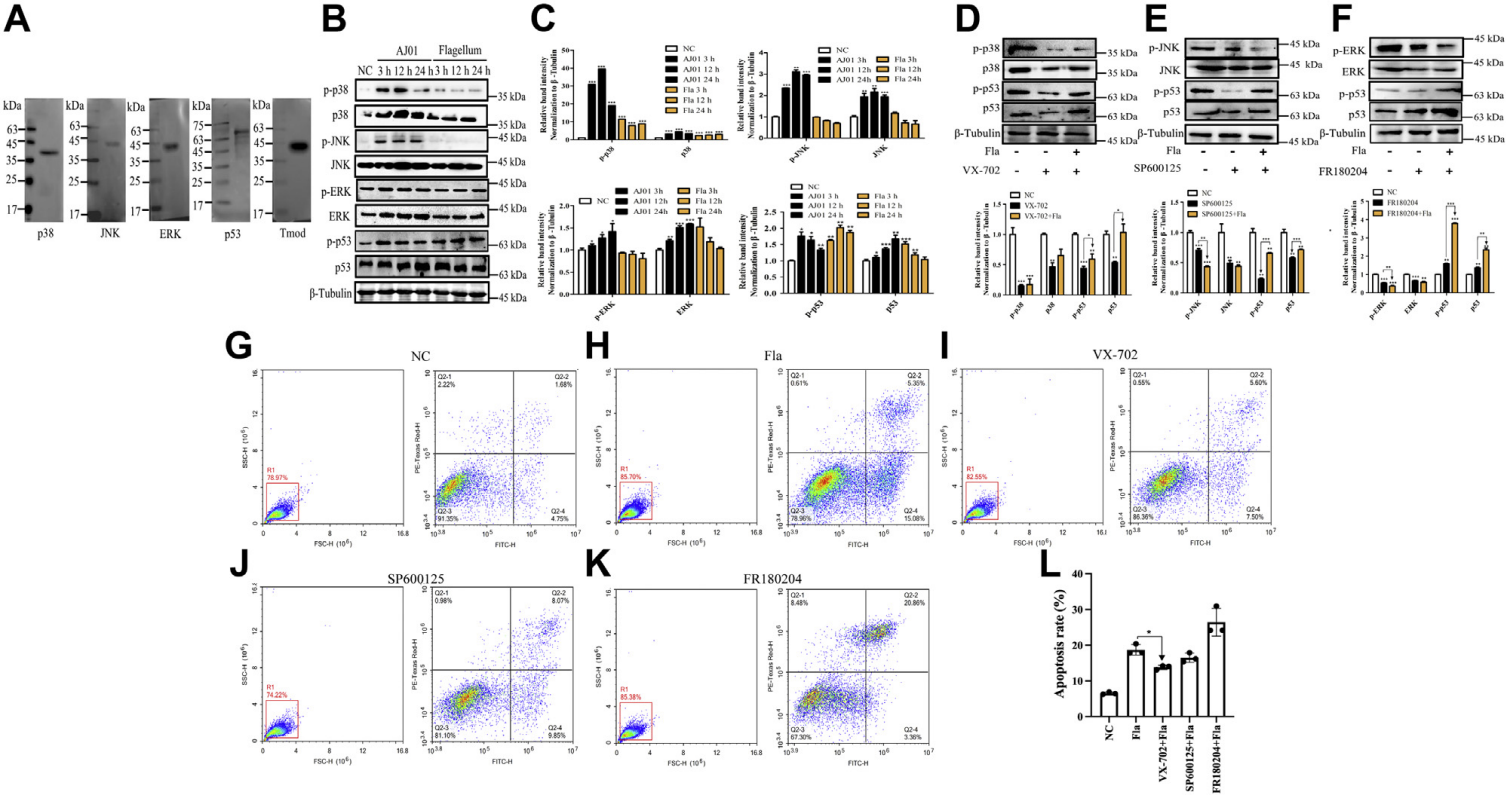
**FliC/AjTmod-mediated coelomocyte apoptosis is dependent on the p38-MAPK pathway.***A*, Western blot analysis of the specificity of p38, JNK, ERK, p53, and Tmod antibodies. *B*, protein abundances and phosphorylation levels of p38, JNK, ERK, and p53 at 3 h, 12 h, and 24 h after AJ01 infection (10^7^ CFU/ml) and flagellum stimulation (100 μg). p38 protein and phosphorylation expression in coelomocytes was determined using p38-labeled mouse polyclonal antibody and phospho-p38 (Thr180/Tyr182) polyclonal antibody. JNK protein expression and phosphorylation in coelomocytes was determined using a JNK-labeled rabbit monoclonal antibody and phospho-JNK (Thr183/Tyr185) monoclonal antibody. ERK protein expression and phosphorylation in coelomocytes was determined using ERK1/2-labeled rabbit monoclonal antibody and phospho-ERK1/2 (Thr202/Tyr204) monoclonal antibody. p53 protein expression and phosphorylation in coelomocytes was determined using p53-labeled mouse monoclonal antibody and phospho-p53 (Ser15) polyclonal antibody, and β-Tubulin-labeled rabbit antibody served as the control. *C*, band density was quantified using ImageJ, and phosphorylation and protein levels of p38, JNK, ERK, and p53 were quantified and normalized to β-Tubulin. Data (means ± SD) are representative of at least three experiments. *Asterisks* indicate significant differences (∗*p*< 0.05, ∗∗*p*< 0.01, ∗∗∗*p*< 0.001). *D*–*F*, screening of the MAPK pathways in response to flagella activation. The coelomocytes were treated with VX-702 (p38 inhibitor, 20 nM), SP600125 (JNK inhibitor, 90 nM), and FR180204 (ERK inhibitor, 0.2 μM), and then 2 μl of flagellum (2.5 μg/μl) was added to each well with inhibitor-treated coelomocytes. The *first column* of the *upper panels* presents coelomocytes treated with the same volume of PBS for 12 h as the control. The *second column* of the *upper panels* presents coelomocytes treated with inhibitors for 12 h. The *third column* of the *upper panels* presents coelomocytes treated with 5 μg flagellum for 12 h after treatment with inhibitors. The antibodies described above were used to detect the protein expression and phosphorylated of p38, JNK, ERK, and p53. Band density was quantified using ImageJ in the *lower panels* as described above. *G*–*L*, the apoptosis rate of cells treated with different inhibitors was detected as previously described. Data are the means of three independent experiments and presented as the means ± SD. FliC, flagellin C.

The MAPK family is reported to be induced and activated by the flagellin present in many bacterial species, including *S. typhimurium*, *Streptococcus enteritidis*, *Streptococcus aureus*, and *Yersinia pestis* (46, 47, 48). To elucidate the molecular mechanism behind FliC/AjTmod-mediated coelomocyte apoptosis, we assessed the protein and phosphorylation levels of the mediators of three MAPK signaling pathways (p38, JNK, and ERK) after AJ01 and flagellum treatments. The p38, JNK, ERK, p53, and Tmod antibodies displayed high specificity to the total protein from *A. japonicus* coelomocytes by Western blot analysis (Fig. 6*A*). Further immunoblot analysis showed that the protein abundance and phosphorylation levels of p38, JNK, and ERK were all significantly increased in the AJ01 challenge groups. However, only the protein abundance and phosphorylation levels of p38 were significantly higher in the flagellum treatment group than the control group (Fig. 6, *B* and *C*). We further treated coelomocytes with different MAPK inhibitors for 12 h, including inhibitors VX-702 for p38 (20 nM), SP600125 for JNK (90 nM), and FR180204 (0.2 μM) for ERK, before flagellum treatment. The three specific inhibitors all significantly decreased the protein abundance and phosphorylation levels of p38, JNK, and ERK. Under this condition, further flagellum treatment could not lead to higher protein abundance and phosphorylation levels of p38 (Fig. 6, *D*–*F*) but led to significantly lower coelomocyte apoptosis levels than those of the control group (Fig. 6, *G*–*I*). There were no changes in the levels of coelomocyte apoptosis in the JNK and ERK inhibitor treatment groups (Fig. 6, *J* and *K*). These results suggested that the AJ01 flagellum promotes coelomocyte apoptosis through the p38-MAPK pathway.

### FliC/AjTmod-p38-MAPK–induced coelomocyte apoptosis requires p53 activation

**Figure 7:**
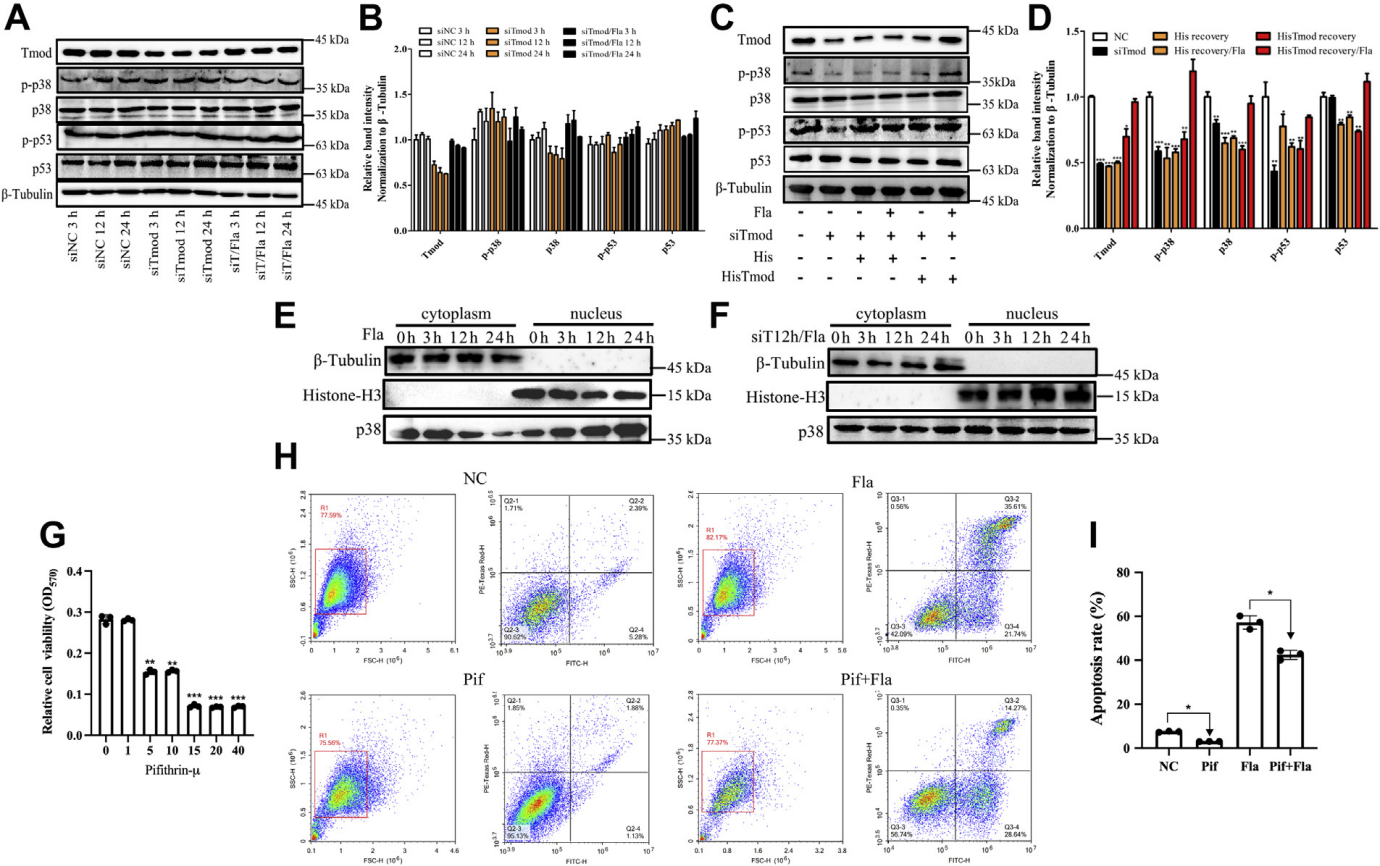
**FliC/AjTmod-p38-MAPK-induced coelomocyte apoptosis requires p53 activation.***A*, Western blotting analysis of p38 and p53 protein expression and phosphorylation profiles in coelomocytes post siNC transfection (20 μM; 3 h, 12 h, and 24 h), siTmod transfection (20 μM; 3 h, 12 h, and 24 h) and flagellum treatment after 12 h of siTmod transfection (100 μg; 3 h, 12 h, and 24 h). *B*, band density of (*A*) was quantified using ImageJ. *C*, Western blotting analysis of p38 and p53 protein and phosphorylation expression profiles in coelomocytes post siNC transfection (20 μM; 12 h), siTmod transfection (20 μM; 12 h), siTmod+His tag recovery (100 μg; 12 h), siTmod+His tag recovery+flagellum treatment (100 μg; 12 h), siTmod+HisTmod recovery (100 μg; 12 h), and siTmod+HisTmod recovery+flagellum treatment (100 μg; 12 h). *D*, band density of (*C*) was quantified using ImageJ. *E*, nuclear protein level of p38 after 3 h, 12 h, and 24 h in the 100 μg flagellum treatment. After flagellum treatment, the collected coelomocytes were used to extract nuclear proteins, and the protein level of p38 was detected by Western blotting analysis. *F*, nuclear protein level of p38 at 3 h, 12 h, and 24 h in the 100 μg flagellum treatment at 12 h after siTmod transfection. Under the above conditions, the collected coelomocytes were used to extract nuclear proteins, and the protein level of p38 was detected by Western blotting analysis. β-Tubulin antibody was used as a cytosolic marker. Histone-H3 was used as a nuclear marker. *G*, the optimal concentration of the p53 inhibitor pifithrin-μ was detected by the MTT Cell Proliferation and Cytotoxicity Assay Kit. Sea cucumber coelomocytes were cultured in 96-well plates at more than 5000 cells per well, and different concentrations of pifithrin-μ were added. After the addition of 10 μM MTT (28 °C for 4 h) and 100 μM formazan solution (37 °C for 4 h), the absorbance value was measured at 570 nm. *H* and *I*, coelomocyte apoptosis detection after flagellum treatment (Fla, 5 μg, 12 h), pifithrin-μ treatment (Pif, 1 μM, 12 h), and flagellum treatment after pifithrin-μ treatment (Pif+Fla). The X and Y axes represent PI and Annexin V, respectively. The cells in the *red box* indicate a portion of all examined cells. Data are the means of three independent experiments and are presented as the means ± SD. FliC, flagellin C; siTmod, siRNA targeting AjTmod. ∗*p* < 0.05, ∗∗*p* < 0.01, ∗∗∗*p* < 0.001.

Previous studies have shown that phosphorylated p53 is one of the key regulators involved in the p38-MAPK–mediated apoptosis pathway (49, 50, 51, 52, 53). Thus, we further determined whether p53 phosphorylation was necessary for p38-MAPK–mediated coelomocyte apoptosis in sea cucumbers. As shown in Figure 6, *B* and *C*, both the protein abundance and phosphorylation levels of p38 and p53 were found to be significantly increased after AJ01 or AJ01 flagellum challenge. Moreover, VX-702 treatment significantly decreased the protein abundance and phosphorylation levels of p53 (Fig. 6*D*) and reduced coelomocyte apoptosis with a 5% reduction (*p* < 0.05) (Fig. 6*L*). To further elucidate the activation of p38 and p53 depending on AjTmod, the protein abundance and phosphorylation levels of p38 and p53 were analyzed under the conditions described in Section “RNA silencing and recovery assay.” The results showed that both the siTmod and siTmod+flagellum groups failed to promote an increase in the protein abundance and phosphorylation levels of p38 and p53 (Fig. 7, *A* and *B*). Rescue experiments performed by adding rTmod recovered the protein abundance and phosphorylation levels of p38 and p53, whereas His tag protein administration did not restore their expression levels (Fig. 7, *C* and *D*).

p38 usually shifts from the cytoplasm to the nucleus to activate the expression of transcription factors in response to external stimulation (53). Therefore, we further assessed the effect of the flagellum- or AjTmod-mediated nucleation process of p38. β-Tubulin was used as the cytoplasmic marker, and histone-H3 was used as the nuclear marker. As shown in Figure 7*E*, relative to that in the control, the nuclear protein level of p38 was significantly increased after flagellum treatment. However, the nuclear protein level of p38 showed no significant difference after siTmod transfection (Fig. 7*F*). These results suggested that p38 activates p53 through nuclear translocation in *A. japonicus*.

### p53 is a proapoptotic factor in *A. japonicus*

Although p53 has been shown to participate in diverse cellular processes, including cell cycle regulation, cellular senescence, DNA repair, cell differentiation, and angiogenesis (54), the most important property of p53 is based on its function in apoptosis because it can selectively destroy stressed or abnormal cells and protect the organism from diseases (55). To understand the apoptosis function of p53, a specific p53 inhibitor, pifithrin-μ (Selleck), was used to suppress p53 activity. An MTT Cell Proliferation and Cytotoxicity Assay Kit (Beyotime) was used to determine the optimal concentration of pifithrin-μ, and 1 μM was used as our optimal inhibitory concentration for p53 functional analysis (Fig. 7*G*). As shown in Figure 7*H*, after inhibition by pifithrin-μ, coelomocyte apoptosis was significantly lower than that in the control group. To further determine whether flagellum-regulated apoptosis was dependent on p53, apoptosis was assayed in flagellum- and pifithrin-μ+flagellum-treated coelomocytes. The results showed that the level of coelomocyte apoptosis in the pifithrin-μ+flagellum-treated group was significantly lower than that of the flagellum-treated group by 10% (Fig. 7, *H* and *I*).

## Discussion

**Figure 8:**
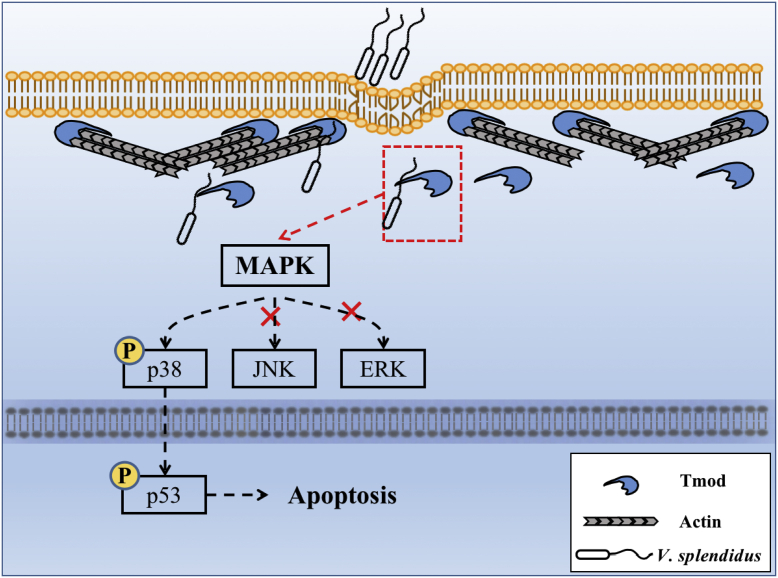
**Schematic of the role of FliC in promoting *coelonocyte apoptosis* by binding AjTmod.** Following AJ01 infection, Tmod bound to the internalized AJ01 flagellum component FliC based on recognition through the LRR domain and then promoted p38 phosphorylation. Phosphorylated p38 entered the nucleus to activate p53 expression, which directly modulated coelomocyte apoptosis. FliC, flagellin C.

Bacterial FliC is a principle immunogenic determinant that regulates various immune responses by binding to PRRs, including TLR5 and NLRs (17, 18, 56, 57). However, the functional role of FliC from *V. splendidus* AJ01, the major pathogen for skin ulcer syndrome in *A. japonicus*, is largely unknown. In this study, we confirmed that the injected AJ01 flagellum can cause *A. japonicus* coelomocyte apoptosis and tissue structure damage (Fig. 2). Damage to the outer skin and tissues might be attributed to coelomocyte migration in response to flagellum challenge. In our previous work, we confirmed that coelomocytes could migrate to the body wall in response to *V. splendidus* infection *via* different cytokines (58, 59). In addition, sea cucumber is an invertebrate with an open vascular system, and the injected flagellum can also directly affect other tissues. However, the typical FliC membrane receptor TLR5 was absent in the *A. japonicus* genome, and only TLR1 and TLR3 were present (60). Although NLRC4 could be detected in *A. japonicus*, it served as a membrane protein without LRR and CARD domains (61), different from the typical common cytoplasmic NLRs. All this evidence suggests that the AJ01 flagellum regulates the sea cucumber immune response by binding a novel receptor. Fortunately, the novel FliC intracellular interactive protein AjTmod with an LRR domain was identified from sea cucumber coelomocytes, in which the LRR domain was found to be a key for binding FliC. Further functional analysis elucidated that FliC/AjTmod mediated coelomocyte apoptosis by promoting p38 translocation into the nucleus to activate p53 expression (Fig. 8). These findings provide the first evidence that AjTmod serves as a bacterial FliC-interacting protein in regulating coelomocyte apoptosis, rather than as a cytoskeletal protein.

"Altruistic death" is a defense mechanism employed by host cells that sometimes deprives the protective niche of intracellular pathogens and exposes them to other components of the immune system (62). In the face of many obligate and facultative intracellular pathogens, the host cells undergo apoptosis, which is the dominant form of cell death during organismal development and homeostasis (63). At present, many intracellular pathogens have been reported to induce host cell apoptosis, and the methods and pathways of apoptosis induced by different pathogens are different (64). *Streptococcus pneumoniae-* and *Francisella tularensis*-induced macrophage apoptosis is associated with intracellular bacterial load. After the intracellular bacteria were killed by antibiotics, the level of apoptosis was found to also decrease significantly, which indicates an apoptosis induction mode closely related to the intracellular bacterial load (65, 66). Some intracellular pathogens induce apoptosis by secreting effector factors, such as *Shigella flexneri*, *S. typhimurium*, and *Listeria monocytogenes*. *S. flexneri* and *S. typhimurium*, *via* a type III secretion system and invade plasmid antigen B (67, 68, 69) and invade plasmid antigen B homolog (SipB) (70, 71, 72), can directly bind and activate caspase-1, resulting in apoptosis. *L. monocytogenes* secretes the pore-forming toxin listeriolysin O, which might insert into the mitochondrial membrane, thereby causing the release of cytochrome C, which activates the caspase cascade to induce apoptosis (73, 74, 75). Most of the above pathogens induce apoptosis by TNF-α- or caspase-dependent pathways, although atypical apoptosis pathways are also observed. *Chlamydia psittaci* induces apoptosis in macrophages and epithelial cells that seems to be independent of known caspases, which might be reminiscent of the caspase-independent apoptosis associated with mitochondria (76, 77). During the past decade, many studies have shown that mitochondrial-mediated apoptosis is mostly initiated by p53, and many p53 target genes involved in apoptosis have been identified (78, 79, 80, 81). Similarly, we found that the flagellum of *V. splendidus* AJ01 could induce p53-dependent coelomocyte apoptosis in *A. japonicus*. p53 in *A. japonicus* is activated by AjTmod/FliC and p38-MAPK-p53, which may regulate apoptosis-related molecules through mitochondria and finally activate apoptosis through caspases. Thus, we determined that FliC/AjTmod-p53–mediated apoptosis regulates mitochondrial apoptosis-related genes, such as Bcl-2 and Bax (81, 82). Mitochondrial-dependent apoptosis in sea cucumber is activated by the recognition of intracellular *V. splendidus* FliC by AjTmod, and it may deprive the protected niche of intracellular AJ01 and expose it to other components of the immune system. In this process, the AjTmod that recognizes FliC plays a key role. When AjTmod was inhibited, the ability of sea cucumber to activate apoptosis was also significantly inhibited (Fig. 5). All this evidence supports the involvement of AjTmod in initiating coelomocyte apoptosis to eliminate intracellular *V. splendidus*.

Tmod, as an important component of the cytoskeleton that can inhibit actin depolymerization and maintain the cytoskeleton, plays essential roles in various cellular processes (31). AjTmod has the same conserved LRR domain as that of other PRRs, such as TLRs and NLRs, which provide the molecular basis for binding to PAMPs. In this study, we confirmed that AjTmod could be induced by *V. splendidus* FliC challenge to specifically bind to FliC by different methods (Fig. 3), in which the LRR domain of AjTmod was critical for its interaction with FliC (Fig. 4). In other works, TLR5 and Naip5 both responded to flagellin and interacted with flagellin in their respective LRR domains (16). Taking TLR5 as an example, FliC-bound TLR5 forms a symmetric m-shaped dimer that mediates dimerization of the intracellular Toll/interleukin-1 receptor domains, which is essential for the activation of downstream pathways (83). By modeling the TLR5 structure combined with select point mutations, the FliC recognition site on TLR5 was narrowed to a concave surface in the curve of the modeled LRR solenoid (84). FliC-induced dimer formation involves the amino acid sequence "KLQTLDLRDNALTTIHFIFSI" from the LRRs of TLR5, which are critical for TLR5 activity because mutations in these LRRs result in forms of TLR5 that fail to transcriptionally activate the NF-κB reporter system (85, 86). The AjTmod LRR domain sequence "TLKSLNVESN" has a consensus LRR sequence "XLXXLXLXXN" with TLR5 except for the seventh valine. On the other hand, AjTmod contains only two LRR repeat domains unlike the multiple LRR domains of TLR5. Early work showed that the 14 and 12 LRR repeat domains of TLR5 can form stable complexes with FliC for structural analysis, but the six LRR repeat domains cannot (87). TLR5 is mostly localized at the plasma membrane, where it recognizes extracellular FliC. FliC-bound TLR5 transmits signals through the Toll/interleukin-1 receptor domain and connector proteins, such as MyD88, and initiates signaling cascades that lead to the production of proinflammatory cytokines (88). It has also been reported that FliC-bound TLR5 is internalized. FliC-bound TLR5 is also observed in polarized intestinal epithelial cells and antigen-presenting cells and needs to be internalized into lysosomes for degradation (89, 90). In this context, a single AjTmod with few LRRs may not bind stably to FliC. As shown in Fig. 3*A*, the ratios between AjTmod and FliC are not 1:1, but no less than four molecules of FliC may be required to bind one AjTmod, and this finding should be further investigated in our future work.

Tmod on the cytoplasmic surface of plasma membranes is thought to be common to nearly all higher eukaryotic cell types (91, 92). Consistent with these results, the deduced amino acid of AjTmod has no transmembrane region by SMART analysis. AjTmod was also colocalized with inner membrane by immunofluorescence analysis (Fig. S2, *E* and *F*). All these evidences supported that AjTmod was a kind of protein located in the inner membrane and cytoplasm of *A. japonicus* coelomocytes. Intracellular receptor initiation-specific intracellular signals depend on intracellular calcium, kinase activation, and direct interaction with cytoskeletal proteins (93, 94). As an important component of the cytoskeleton, AjTmod should also follow the signal transmission mode of this intracellular receptor. All these findings support that the signal transmission of AjTmod stimulated by FliC is achieved by the internalization of AjTmod into the cytoplasm.

In our work, the acid hydrolysis method was used in this study to extract purified flagellin. The extraction from *Escherichia coli* DH5α without flagella performed with this method served as a control to confirm that potential contamination had no effect on skin syndrome development and the change in coelomocyte apoptosis (data not shown). Based on this result, we found that the AjTmod–FliC interaction signal was transmitted to p38-MAPK (Figs. 6 and 7). p38, as an important member of the MAPK family, can be activated by various environmental stresses and inflammatory cytokines. Typical p38-MAPK activation is achieved by phosphorylation of Thr^180^ by upstream MKK3 and MKK6 (95, 96, 97). In *A. japonicus*, both MKK6 (GenBank no. ASA69193.1) and p38 phosphorylated sites (Ser^207^ and Thr^211^) are all conserved, which implies that MKK6 is likely to be an intermediate sensor for AjTmod to activate p38. A previous study also found that leucine-rich repeat kinase 2 could bind to MKK6 and stimulate MKK6 phosphorylation (98). Further work should investigate the connection among AjTmod, AjMKK6, and Ajp38 in mediating coelomocyte apoptosis.

## Experimental procedures

### Animals and cell culture

The sea cucumbers used in this work were commercially cultured animals, and all experiments were conducted in accordance with the recommendations in the Guide for the Care and Use of Laboratory Animals of the National Institutes of Health. The study protocol was approved by the Experimental Animal Ethics Committee of Ningbo University.

Healthy adult sea cucumbers (116 ± 14 g) were collected from the Dalian Pacific Aquaculture Company and acclimatized in seawater (salinity, 28; temperature, 16 °C) for 3 days. The sea cucumbers from the experimental and control groups were dissected with sterilized scissors on ice, and the coelomic fluids were filtered through a 300-mesh cell cribble and centrifuged at 800*g* for 10 min to harvest the coelomocytes for subsequent gene and protein expression analysis.

*V. splendidus* AJ01 was cultured at 28 °C in 2216E medium consisting of 5 g/L tryptone, 1 g/L yeast extract (Solarbio), and 0.01 g/L FePO_4_ in filtered seawater. *E. coli* DH5α and BL21 (DE3) strains were purchased from Takara Bio and cultured in LB medium at 37 °C.

HeLa cells were cultured at 37 °C in Dulbecco’s modified Eagle’s medium (DMEM) supplemented with 10% fetal bovine serum (Sangon) and under a 5% (vol/vol) CO_2_ atmosphere. Primary coelomocytes from mature *A. japonicus* coelomic fluid were cultured at 28 °C in L-15 medium with 10 U/ml penicillin, 100 μg/ml streptomycin, and 50 μg/ml gentamicin.

### Flagellum extraction

*V. splendidus* AJ01 flagellum was extracted according to the method by Peel *et al*. (99). When the A_600_ of cultured *V. splendidus* AJ01 was 0.6, the bacteria were collected by centrifugation at 5000*g* for 30 min and washed with PBS three times. The collected AJ01 was resuspended in 5 ml PBS and adjusted to pH 2.0 with HCl. The culture was stirred at room temperature for 30 min and centrifuged at 5000*g* for 10 min to collect the supernatant. Saturated (NH_4_)SO_4_ solution was then slowly added to the supernatant at a final concentration of 2.67 mol/l and stirred at 4 °C for 12 h before precipitates were collected by centrifugation at 14,000*g* for 10 min. Finally, the collected flagellum was dissolved in 8 ml PBS. The flagellum solution was added to dialysis membranes (Solarbio) and kept rotating in PBS for 48 h. The concentration of the obtained flagellum was determined with a BCA Protein Assay Kit (Sangon).

### Histological analysis

Sea cucumbers injected with 50 μl extracted flagellum (2 μg/μl) for 24 h served as the AJ01 flagellum challenge group. Sea cucumbers injected with 100 μg BSA with the same volume served as the negative control, and sea cucumbers without any treatment served as the blank control. The tissue samples collected from each specimen were fixed with 10% neutral formaldehyde fixative for 24 h. Then, the fixed tissues were rinsed using 70% alcohol and dehydrated using ethanol at increasing concentrations (70, 85, 90, 95, and 100%). Tissue samples were clarified in xylene, embedded in paraffin wax at an average fusion temperature of 56 °C, and sectioned at 7 μm thicknesses with a microtome (KD-3358). Then, the sections were observed under a microscope (ZEISS Axio Vert. A1).

### Cell apoptotic assay

*In vivo* cell apoptosis functions were assessed by flow cytometry analysis. After sea cucumbers were challenged with AJ01 or the extracted flagella for 24 h, the apoptosis rate of the collected coelomocytes was measured by FACScan (Becton Dickinson Biosciences) using an Annexin-V FITC apoptosis detection kit (Beyotime). Sea cucumbers without any treatment were used as the blank group, and sea cucumbers treated with 2216E or PBS were used as the negative control group. Briefly, the collected coelomocytes were resuspended in 195 μl Annexin V-FITC binding buffer at a final concentration of 10^6^/ml and then mixed with 5 μl Annexin V-FITC and 10 μl PI successively. After the mixture was incubated at 25 °C for 10 min, the samples were analyzed by FACScan.

### Generation of recombinant protein *in vitro*

Total RNA was extracted using RNAiso plus (Takara) according to the supplied protocol. The quality and quantity of RNA of each sample were determined by a NanoDrop 2000. All extracted RNA samples with A260/A280 ratios greater than 1.7 were used for cDNA synthesis. The primers used in this paper are shown in Table 1. FliC and FliC-binding protein were purified and ligated into the pMD19-T simple vector (Takara). The purification of PCR products, the extraction of DNA fragments from agarose gels, and the preparation of plasmids were performed using kits from Omega BioTek (GA) according to the manufacturer’s instructions. Recombinant proteins were induced with 0.2 mM IPTG. The His-tagged proteins were purified using Ni-TED Sefinose (TM) resin and GST-fused protein by GST-Sefinose (TM) Resin. The proteins from the pET28a and pGEX-4T-2 vectors without any insertion were used as control proteins.

**Table 1:** Primer and siRNA sequences used in this study

Names	Sequences (5′-3′)	Application
FliC	GGATCCATGCTGAACCAATCTTTGGAGCGCT CTCGAGTTAACCCAGTAAGGTTAAGGCAAGA	Recombinant protein
GSTFliC	GGATCCATGCTGAACCAATCTTTGGAGCGCT GAATTCTTAACCCAGTAAGGTTAAGGCAAGA	Recombinant protein
AjTmod	GCGGCCGCATGCCGACAGTAACCATGGC CTCGAGTCATTCCTTCTTTGTCCTCT	Recombinant protein
qTmod	TGAAACCGCACCTTATCTCGCAC CTCTGTCTCCACTTGAATACCCAT	Real-time PCR
qβ-Tubulin	GCACATCAAGCCGTCAAACTCAC TATGCCCGCATAGCAAACATACC	Real-time PCR
siTmod-600	CCUCCUUGAAGGAACUAAATT UUUAGUUCCUUCAAGGAGGTT	RNAi
siTmod-1016	CUCAGGAACUAUGAAAUAATT UUAUUUCAUAGUUCCUGAGTT	RNAi
siRNA (NC)	UUCUCCGAACGUGUCACGUTT ACGUGACACGUUCGGAGAATT	RNAi
EGFP-FliC	CTCGAGATGCTGAACCAATCTTTGGA AAGCTTTTAACCCAGTAAGGTTAAGG	Recombinant plasmid
Flag-Tmod	CTCGAGATGCCGACAGTAACCATGGC AAGCTTTCATTCCTTCTTTGTCCTCT	Recombinant plasmid
Flag-TmodTro	CTCGAGAATGAGATGGACCCCGATGA AAGCTTTTGATTCAACATACTGTGGA	Recombinant plasmid
Flag-TmodTLRR	CTCGAGATCTTGGAAGCTCTGGAGAA AAGCTTTCATTCCTTCTTTGTCCTCT	Recombinant plasmid

### Pull-down assay

A pull-down assay was performed to select the potential receptor of *V. splendidus* AJ01 FliC. Purified recombinant His-FliC (rFliC) fusion protein immobilized on His resin was incubated on ice for 30 min and washed with wash buffer for TED Sefinose (TM) Resin (Sangon) three times. Then, 500 μg of total protein from *A. japonicus* coelomocytes was added to prepared rFliC resin and incubated for another 4 h at 4 °C. The resin was washed with wash buffer to remove protein impurities and finally eluted with 250 mM imidazole. The His tag was used as a control. The eluent was detected by SDS–PAGE, and the differential protein fragments were detected by mass spectrometry. Similarly, the reverse pull-down assay was performed with immobilized GST-fused FliC-binding protein as the target, which was incubated with rFliC solution. After washing and elution, the eluent was subjected to Western blotting and SDS–PAGE.

### Western blotting

Antiserum for FliC, FliC-binding protein, and internal control β-Tubulin were prepared according to protocols reported in our previous work (100, 101, 102). Other antibodies used in this study are shown in Table 2. A total of 50 μg of protein from each sample was separated by SDS–PAGE before transfer to a 0.45-mm pore nitrocellulose membrane with an ECL Semidry Blotter (Amersham Biosciences). The membrane was blocked with 5% skim milk in TBST (20 mM Tris–HCl, 150 mM NaCl, and 0.05% Tween-20) at 37 °C for 1 h. The membranes were incubated with diluted polyclonal antibodies diluted to 1:400 in 5% skimmed milk at 4 °C for 12 h. After washing the membrane three times with TBST for 10 min each, the membrane was subsequently incubated with diluted goat-anti-mouse or goat-anti-rabbit IgG (Sangon) diluted to 1:3000 at room temperature for 1 h. The membrane was washed and incubated in Western Lightning-ECL substrate (PerkinElmer) prior to exposure to X-OMAT AR X-ray film (Eastman Kodak).

**Table 2:** Antibodies information in this study

Antibodies	Isotype	Use	Product no.	Source
Phospho-p38 MAPK (Thr180/Tyr182)	Rabbit	WB:(1:1000)	28796-1-AP	Proteintech
JNK Monoclonal Antibody	Rabbit	WB:(1:1000)	1A12E1	Proteintech
Phospho-JNK1/2 (Thr183/Tyr185)	Rabbit	WB:(1:500)	AF5860	Beyotime
ERK1/2 Rabbit Monoclonal Antibody	Rabbit	WB:(1:500)	AF1051	Beyotime
Phospho-ERK1/2 (Thr202/Tyr204)	Rabbit	WB:(1:1000)	80031-1-RR	Proteintech
Phospho-p53 (Ser15) Polyclonal Antibody	Rabbit	WB:(1:1000)	28961-1-AP	Proteintech
p53 Monoclonal Antibody	Rabbit	WB:(1:1000)	60283-2-Ig	Proteintech
Beta Tubulin Polyclonal Antibody	Rabbit	WB:(1:1000)	10068-1-AP	Proteintech
Histone-H3 Polyclonal Antibody	Rabbit	WB:(1:1000)	17168-1-AP	Proteintech
His-Tag (2A8) Antibody	Mouse	WB:(1:5000) IP:(1:200)	M20001	Abmart
Anti-GST Tag monoclonal antibody	Mouse	WB:(1:2000) IP:(1:100)	D190101	Sangon Biotech
Flag-Tag Antibody	Mouse	WB:(1:5000) IP:(1:200)	TT0003	Abmart
GFP Antibody	Mouse	WB:(1:5000) IP:(1:200)	P30010	Abmart
p38	Mouse	WB:(1:200)		Antiserum
FliC	Mouse	WB:(1:200)		
Tmod	Mouse	WB:(1:200)		

### Microscale thermophoresis assay

The affinity of the purified GSTFliC to FliC-binding protein was measured using the Monolith NT.115 with GST protein as the control (Nanotemper Technologies). FliC-binding protein with a His tag was fluorescently labeled according to the manufacturer’s procedure. The solution buffer was exchanged with labeling buffer, and the protein concentration was adjusted to 10 μM. Then, the fluorescent dye NT-647-NHS was added, mixed, and incubated for 30 min at 25 °C in the dark. Finally, the labeled proteins were dialyzed with column B (Nanotemper L001) and eluted with 50 mM Tris–HCl (pH 8.0) supplemented with 0.02% Tween 20. For each assay, the labeled protein (approximately 5 μM) was incubated with the same volume unlabeled HisTmod of 16 different serial concentrations in 50 mM Tris–HCl (pH 8.0) supplemented with 0.02% Tween 20 at room temperature for 10 min. The samples were then loaded into silica capillaries (Polymicro Technologies) and measured at 25 °C by using 20%-40% LED power and 20% microscale thermophoresis power. Each assay was repeated three times. Data analyses were performed using Nanotemper analysis software MO.Affinity Analysis.

### Far-western assay

The far-western assay was performed according to protocols reported in a previous work (103). Briefly, 5 μg, 10 μg, and 20 μg of rFliC-binding protein and 20 μg of rHis-tag were separated by SDS–PAGE and transferred to a 0.45-mm polyvinylidene fluoride membrane. The membrane was washed with denaturation buffer (6 M guanidine-HCl in basic buffer [20 mM Hepes (pH 7.5), 50 mM KCl, 10 mM MgCl_2_, 1 mM DTT, 0.1% Triton X-100, 5% glycerol]) at 4 °C for 10 min with gentle agitation. Half of the volume of the denaturation buffer was replaced with basic buffer, and the wash was continued for another 10 min. Serial dilution and washing were performed five more times until the concentration of guanidine-HCl reached 90 mM. Then, basic buffer was used to wash the membrane for 10 min. The membrane was blocked with 5% skim milk in TBST at 37 °C for 1 h and then incubated in TBST containing 1 μg/μl rFliC. Similarly, 5 μg, 10 μg, 20 μg rFliC and 20 μg rGST were separated by SDS–PAGE and transferred to a polyvinylidene fluoride membrane, and then the membrane was incubated with 1 μg/μl rFliC-binding protein. After rotation at 4 °C overnight, TBST was used to wash off the noninteracting proteins, and the bound proteins were analyzed by Western blotting, as described above.

### ELISA analysis

The 96-well plates used for ELISA were blocked with 5% BSA in PBS at 37 °C for 3 h. After washing three times with PBS containing 0.05% Tween 20 (PBST), 0.01 μg, 0.1 μg, 1 μg, and 10 μg rFliC or rFliC-binding protein were added to a volume of 100 μl ELISA Coating Buffer (Solarbio) and incubated overnight. After washing three times with PBST, 600 ng rFliC-binding protein or rFliC was added and incubated for 3 h. After washing three times with PBST, 100 μl of the GST-labeled antibody (Sangon) dilution (1:1000 in PBS) was added to each well and incubated at 37 °C for 1 h. After washing, the wells were treated with 1:3000 diluted goat-anti-mouse or goat-anti-rabbit IgG (Sangon) at 37 °C for 1 h. After the last three washes, a TMB Kit (Solarbio) was used for color development, and 50 μl of hydrochloric acid (1 M) was added to terminate the reaction. The absorbance of the developed color was read at 450 nm with a UV–Vis spectrophotometer (Beckman).

### Immunofluorescence

Immunofluorescence analysis was performed to detect the location of the FliC-binding protein and FliC. The primary coelomocytes cultured overnight on slides were fixed with 4% paraformaldehyde for 30 min. Coelomocytes were permeabilized with 0.5% Triton X-100 for 20 min, and the other group was not treated with the permeabilization solution. The slide was blocked with 5% BSA in PBST for 30 min and then incubated with diluted polyclonal antibodies diluted to 1:400 in 5% PBST at 37 °C for 1 h. After washing the slide three times with PBST for 10 min each, the membrane was subsequently incubated with goat-anti-mouse Alexa Fluor 488 (Beyotime) diluted to 1:800 at 37 °C for 1 h. The slide was washed and stained with DAPI (Beyotime) and sealed with antifade mounting medium (Beyotime). Finally, the slide was visualized using a laser scanning spectral confocal microscope (TCS SP2; Leica).

### Coimmunoprecipitation assay

Different fragments of *Tmod* (1–1065), *TTro* (109–444), and *TLRR* (664–1065) were inserted into the pcDNA3.1-Flag vector. The fragment of *fliC* was inserted into the pIRES2-EGFP vector. Flag-*Tmod*/Flag-*TTro*/Flag-*TLRR* (8 μg)- and EGFP-*fliC* (8 μg)-expressing vectors and a 10 μl Lipo6000 mixture were cotransfected into HeLa cells in a 10 cm dish with 6 ml medium. For immunoprecipitation, the cells were lysed on ice using RIPA lysis buffer containing 50 mM Tris (pH 7.4), 150 mM NaCl, 1% NP-40, 0.5% sodium deoxycholate, and 0.1% SDS. Protein A + G agarose (Beyotime) was washed with RIPA lysis buffer three times. The supernatant was collected and incubated with 30 μl Protein A + G agarose. Four hours later, the beads were immunoblotted with either GFP- (Beyotime) or Flag-labeled antibodies (Beyotime). The proteins were detected by Western blot. The lysate, pcDNA3.1-Flag, and pIRES2-EGFP were used as controls.

### AJ01-challenged sea cucumber

A total of sixty healthy adult *A. japonicus* were equally acclimatized in four tanks for 3 days before conducting the experiment. In brief, one tank without any treatment served as a control. The AJ01-challenged tanks were immersed with a final concentration of 10^7^ CFU/ml AJ01. In the flagellum-treatment group, the purified FliC was resolved in 5 ml PBS as stock solution (2.5 μg/μL). Then, 0.6 μL, 6 μL, and 60 μL stock solution were collected to obtain 100 μl working solution in PBS for challenge. Sea cucumbers without any treatment were used as the blank group, and those injected with 100 μl PBS were used as the negative control group. The coelomic fluids were collected at 0 h, 6 h, 12 h, 24 h, and 48 h postinfection, and then the collected fluids were centrifuged at 800 g at 4 °C for 5 min to harvest the coelomocytes. The collected coelomocyte samples were used for real-time quantitative PCR and protein expression analysis.

Total RNA from the coelomocytes was isolated with an RNAiso Plus kit (Takara), and cDNA was synthesized using the miScript Reverse Transcription Kit (Qiagen). qPCR was performed on an Applied Biosystems 7500 real-time PCR system.

### RNA silencing and recovery assay

The specific siRNAs for target and control genes were synthesized by GenePharma (China). Detailed sequence information for siTmod and siNC is shown in Table 1. The experimental and control siRNAs were dissolved in RNase-free water to obtain 20 μM working solutions. Ten microliters of the siRNA, 10 μl of Lipo6000 transfection reagent (Beyotime), and 80 μl of PBS were mixed and used as the transfection solution. Sea cucumbers were injected with 100 μl of the transfection solution of siNC or siTmod groups. After transfection for 3 h, 12 h, and 24 h, the collected coelomocyte samples were used for protein expression analysis.

rTmod was used for the rescue experiment. RNA silencing was conducted as described above. For the *in vivo* recovery assay, 40 μl of rTmod (2.5 μg/μl) was injected into sea cucumbers for another 12 h after AjTmod silencing for 12 h. The control group was injected with an equal volume of purified His-tagged protein. The control and experimental groups were further treated with flagellum for an additional 12 h, and the collected coelomocyte samples were used for protein expression analysis. There were three independent replicates for each group.

### MAPK pathway activation analysis

For the *in vitro* assay, VX-702 (p38 inhibitor, Beyotime), SP600125 (JNK inhibitor, Beyotime), and FR180204 (ERK inhibitor, Beyotime) were used to detect the flagellum response of the MAPK pathway. The primary coelomocytes were treated with VX-702, SP600125, and FR180204 at final concentrations of 20 nM, 90 nM, and 0.2 μM for 12 h, and then 2 μl of flagellum (2.5 μg/μl) was added to each well with inhibitor-treated coelomocytes for another 12 h. An equal volume of PBS solution served as the control group. The collected coelomocyte samples were used for protein expression analysis. For the *in vivo* experiment, the same inhibitor was injected into live sea cucumber (108 ± 12 g) and then treated with flagellum. Coelomocyte apoptosis was assayed according to Section “Cell apoptotic assay.”

### Statistical analysis

Statistical analyses were performed using GraphPad Prism (GraphPad Software). All data are representative of at least three independent experiments and presented as the mean ± SD. For Western blotting, the band density was analyzed with ImageJ, and the protein expression levels were normalized to β-tubulin. Statistical significance was defined as ∗*p* < 0.05, ∗∗*p* < 0.01, ∗∗∗*p* < 0.001.

## Data availability

All data relevant to the study are included in the article or uploaded as supplementary information. Further data is available from the corresponding author at lichenghua@nbu.edu.cn upon reasonable request.

## Supporting information

This article contains supporting information.

## Conflict of interest

The authors declare that they have no conflict of interest with the contents of this article. The *A. japonicus* were commercially cultured animals, and all the experiments were conducted in accordance with the recommendations in the Guide for the Care and Use of Laboratory Animals of the National Institutes of Health. The study protocol was approved by the Experimental Animal Ethics Committee of Ningbo University, China.
