# Supplementary material for: Vibrio splendidus flagellin C binds tropomodulin to induce p38 MAPK-mediated p53-dependent coelomocyte apoptosis in Echinodermata
Source: J Biol Chem. 2022 May 30;298(7):102091. doi: 10.1016/j.jbc.2022.102091 (PMC9249833; doi:10.1016/j.jbc.2022.102091)
Supplement: Supplemental figures [file mmc1.doc]

**Fig. S1**


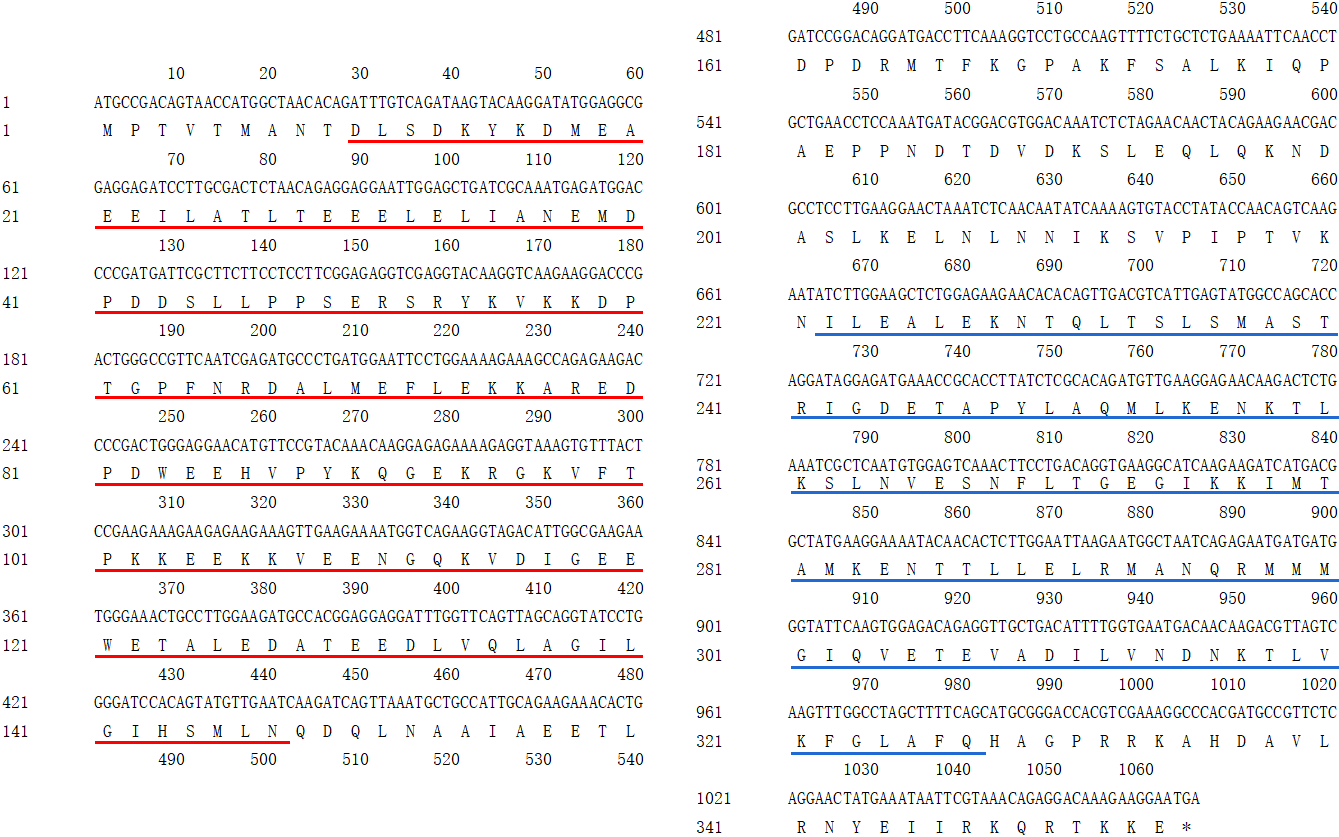


**Fig. S1 Nucleotide and cDNA-derived aa sequences of AjTmod.** Nucleotides and amino acids of AjTmod ORFs shown in capital letters. The predicted conserved tropomodulin domain (37 – 444 aa) of AjTmod is underlined in red. The predicted conserved LRR domain (664 – 984 aa) of AjTmod is underlined in blue.

**Fig. S2**

**Fig. S2** **Temporal expression and localization analysis of AjTmod.** **(A)** *AjTmod* mRNA expression levels in different tissues of *A. japonicus* detected by qRT-PCR. *AjTmod* transcript levels in the body wall, muscle, intestines, tentacles and respiratory trees were normalized to those of coelomocytes. **(B) (C)** *AjTmod* mRNA expression post *V. splendidus* AJ01 infection (10^7^ CFU/mL) and different concentrations of flagellum stimulation. Values are shown as the mean ± SD, n = 3. **(D)** AjTmod protein expression post *V. splendidus* AJ01 infection and flagellum stimulation (100 µg). **(E)** Assay of AjTmod localization by immunofluorescence. Representative confocal micrograph of coelomocyte nuclei stained with DAPI (blue), coelomocyte membranes stained with Dil (red) and AjTmod stained with AjTmod-labeled antibody and Alexa Fluor 488-labeled goat anti-mouse IgG (green). Immunofluorescence was divided into a coelomocyte-permeabilized group with 0.5% Triton X-100 and a nonpermeabilized group without 0.5% Triton X-100. Scale bars, 5 µm. **(F)** Assay of AjTmod localization by western blotting analysis. The sea cucumber coelomocyte membrane and cytoplasmic proteins were extracted with a membrane and cytoplasmic protein extraction kit. Fifty micrograms of protein with different components was used for the western blotting analysis.
